# Lock-Jaw of Infants

**Published:** 1884-12

**Authors:** 


LOCK-JAW OF INFANTS (trismus nascentium) or Nine Days Fits, Crying Spasms, etc.; Its history, Cause, Prevention and Cure. By J. F. Hartigan, M D., Washington, D. C., member of the American Medical Association, etc. New York: Birmingham & Co., 28 Union Square; 14 Cockspur st., Pall Mall, London, 1884.

We are indebted to the author for a copy of this treatise, reviving the views of Dr. Sims as to the cause and treatment of trismus nascentium.

While impartiality is shown in the quotations of authors giving diverse explanations of the probable origin of the affection, cases are presented of the pressure of the occipital bone serving as the exciting cause of the disturbance. In some of the cases detailed, the reader will find a *non sequitur* from the facts presented, and that other cases are not properly classified under this heading, while the general impression produced is that of attempting to sustain a preconceived theory.

The favorable results which are claimed from placing the child in a position to relieve pressure upon the occipital bone are so striking as to commend this proceeding to the attention of mothers and nurses, and to receive the consideration of all physicians. Whether the same explanation is accepted or not, the fact must be recognized if we allow due weight to the testimony of a colleague, who has evidently devoted much observation to this class of cases.

As the profession thus far has discovered no course of treatment which can be relied upon to cure this affliction, it is highly important to avail ourselves of any means of prevention, and certainly "if it does no good it will do no harm" to adopt this suggestion of placing the child upon the side, instead of allowing it to rest upon the back of the head, and also to correct any irregularity in the relations of the occipital to the parietal bones of the new-born infant.

